# Expression of Osteopontin in oesophageal squamous cell carcinoma

**DOI:** 10.1038/sj.bjc.6603296

**Published:** 2006-08-01

**Authors:** Y Kita, S Natsugoe, H Okumura, M Matsumoto, Y Uchikado, T Setoyama, T Owaki, S Ishigami, T Aikou

**Affiliations:** 1 Department of Surgical Oncology and Digestive Surgery, Field of Oncology, Course of Advanced Therapeutics, Graduate School of Medical and Dental Science, Kagoshima University, Sakuragaoka 8-35-1, Kagoshima 890-8520, Japan

**Keywords:** osteopontin, oesophageal cancer, immunohistochemical staining, prognosis, lymph node metastasis

## Abstract

Osteopontin is a multifunctional 34 kDa extracellular matrix protein with a cell-binding domain. It is involved cell adhesion and cell migration and is therefore considered to influence tumorigenesis and/or metastasis. The purpose of the present study was to evaluate the clinical significance of Osteopontin expression in oesophageal squamous cell carcinoma (ESCC). In the present study, we immunohistochemically investigated the relationship between Osteopontin expression and clinicopathological factors including prognosis in surgical specimens of primary tumours in 175 patients with ESCC. Osteopontin was expressed in 48% of 175 patients. Osteopontin expression was significantly correlated with lymph node metastasis, lymphatic invasion, and stage (*P*=0.0015, 0.037 and 0.033, respectively). Tumours with expressing Osteopontin exhibited more lymph node metastasis, lymphatic invasion and advanced stage than the tumour with negative Osteopontin expression. Five-year survival rate was better in patients with negative Osteopontin expression than in those with positive Osteopontin expression (*P*=0.035). However, multivariate analysis revealed that Osteopontin expression was not an independent prognostic factor. As our findings suggest that Osteopontin may play an important role in progress of ESCC, the evaluation of Osteopontin expression is useful for predicting the malignant properties of ESCC.

Osteopontin is a 34 kDa extracellular matrix protein with a cell-binding domain (Sodek *et al*, 2000) and was originally identified as a major component of the noncollagenous organic bone matrix. It secretes adhesive glycoprotein and seems to be involved in osteoblast differentiation, bone formation and remodelling of mineralised tissue (Reinholt *et al*, 1990; Giachelli and Steitz, 2000). Other molecules which share this domain include fibronectin, vitronectin and a variety of other extracellular proteins that bind members of the integrin family of cell surface receptors (Varner and Cheresh, 1996). The expression of Osteopontin has subsequently been demonstrated in a wide range of normal human tissue and body fluid such as osteoblasts, arterial smooth muscle cells, leukocytes, activated macrophages and T cells (Coppola *et al*, 2004), and epithelia of the gastro-intestinal tract (Brown *et al*, 1992; Brown *et al*, 1994). It is a multifunctional protein involved in cell adhesion and cell migration, and has been shown to play important roles in tumorigenesis, tumour invasion, metastasis and prognosis among patients with breast (Tuck *et al*, 1998), lung (Zhang *et al*, 2001; Donati *et al*, 2005), prostate (Thalmann *et al*, 1999), gastric (Ue *et al*, 1998) and colon cancer (Agrawal *et al*, 2002).

Oesophageal squamous cell carcinoma (ESCC) is one of the most aggressive carcinomas in the gastrointestinal tracts. Studies of various biological factors affecting the malignant potential of ESCC have been performed. However, the expression of Osteopontin in ESCC has not been evaluated. The aims of this retrospective study were to examine the expression of Osteopontin in surgical specimens of ESCC and to evaluate whether this is useful in predicting outcome.

## MATERIALS AND METHODS

### Patients and specimens

Subjects were 175 patients with ESCC (160 men and 15 women) who underwent oesophagectomy with lymph node dissection between 1987 and 1998 at Kagoshima University Hospital, Japan. The median age of the patients was 64 years (range 36–83 years). None of these patients underwent endoscopic mucosal resection, palliative resection, preoperative chemotherapy and/or radiotherapy, and none of them had synchronous or metachronous cancer in other organs. Specimens of cancer tissues and noncancerous adjacent tissue were collected from patients after informed consent had been obtained in accordance with the institutional guidelines of our hospital. Using the tumour node metastasis classification of the International Union Against Cancer (Sobin and Fleming, 1997), all of the M1 tumours exhibited distant lymph node metastases. Clinicopathologic data of patients in this study were shown in Table 1. All patients were followed-up after discharge, with X-ray examination and tumour marker assays (squamous cell carcinoma antigen and carcinoembryonic antigen) every 1–3 months, computed tomography every 3–6 months, and ultrasonography every 6 months. Bronchoscopy and endoscopy were performed when necessary. Postoperative follow-up data were obtained from all patients, with a median follow-up period of 28 months (range, 1–175 months).

### Immunohistochemical staining and evaluation

Specimens were cut into 3-*μ*m-thick sections, which were mounted on glass slides. Immunohistochemical staining was carried out using the avidin–biotin–peroxidase complex method (Vectastatin Elite ABC Kit; Vector, Burlingame, CA, USA), following the manufacturer's instructions. Briefly, the immunostaining was performed manually at room temperature. Sections were deparaffinised in xylene and dehydrated in ethanol, endogenous peroxidase activity was blocked by incubating sections for 10 min in 3% hydrogen peroxide in methanol. Sections then were then heated using an autoclave in a citrate buffer (0.01 mol l^−1^, pH 6.5) at 121°C for 15 min to reveal the antigen. After cooling, sections were preincubated in 1% borine serum albmin (BSA) for 20 min. Next, sections were incubated with antiosteopontin monoclonal antibody (1 : 50, Osteopontin, Novocastra Laboratories Ltd, Newcastle, UK) for 60 min. After rinsing with phosphate-buffered saline (PBS) for 15 min, sections were incubated with secondary antibody for 20 min and washed again with PBS for 10 min. Sections were incubated with avidin–biotin complex for 30 min and washed again, and reactions were visualized using diaminobenzidine tetrahydrochloride for 2 min. All samples were lightly counterstained with haematoxylin for 1 min. The negative controls consisted of sections treated with PBS instead of primary antibody. A section of normal tissue of gallbladder was used as positive control. Evaluation of immunohistochemistry was performed independently by two investigators (KY and SN). Positive Osteopontin expression was defined as detectable immunoreaction in perinuclear and other cytoplasmic regions of >10% of the cancer cells. To evaluate expression of Osteopontin, 10 fields (within the tumour and at the invasive front) were selected, and expression in 1000 tumour cells (100 cells/field) was evaluated using high-power (× 200) microscopy.

### Statistical analysis

Statistical analysis of group differences was performed using the *χ*^2^ test. The Kaplan–Meier method was used for survival analysis, and differences in survival were estimated using the log rank test. Prognostic factors were examined by univariate and multivariate analyses (Cox proportional hazards regression model). *P*<0.05 was considered to indicate statistical significance. All statistical analyses were performed using the StatView™ for Windows software (Version 5.0, Abacus Concepts, Berkeley, CA, USA).

## RESULTS

### Expression of Osteopontin in oesophageal squamous cell carcinoma

**Figure 1:**
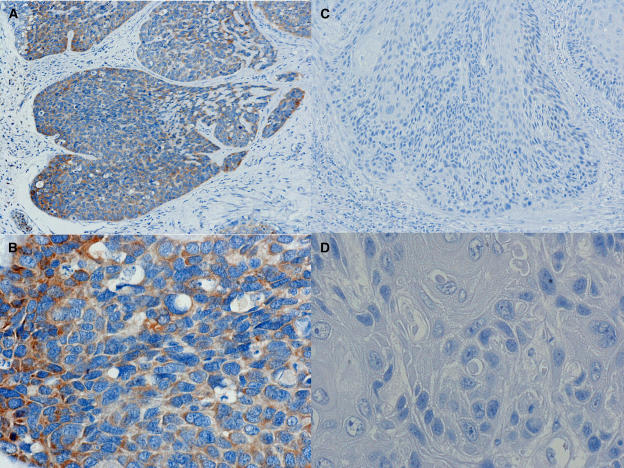
Expression of Osteopontin in ESCCs. (**A**) positive expression of Osteopontin was detectable in cytoplasmic regions (× 100); (**B**) magnified view (× 400) (**C**) negative expression of Osteopontin (× 100). (**D**) magnified view (× 400).

Osteopontin was expressed in the cytoplasm of ESCC cells in 48% (84 of 175) of all patients (Figure 1).

### Relationship between Osteopontin and clinicopathologic variables

Osteopontin expression was significantly associated with the following clinicopathologic parameters: lymph node metastasis, stage and lymphatic invasion (Table 1). Patients with positive Osteopontin expression had more lymph node metastasis and greater lymphatic invasion than those with negative Osteopontin expression (*P*=0.0015 and 0.037, respectively).

### Relationship between Osteopontin expression and prognosis

**Figure 2:**
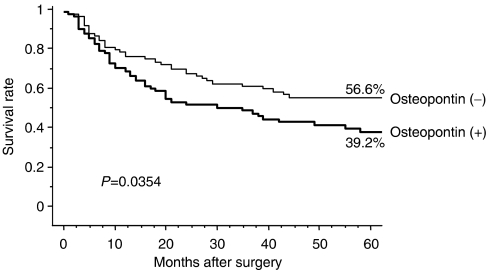
The postoperative 5-years survival curves between the patients expression (−) and expression (+) with Osteopontin. There was a significant difference between the patients with Osteopontin (−) and (+) expression (*P*=0.0354).

The 5-year survival rate was significantly lower in patients with positive Osteopontin expression than in those with negative Osteopontin expression (*P*=0.035; Figure 2).

### Univariate and multivariate analyses of survival

Tables 2 and 3 show univariate and multivariate analyses of factors related to patient prognosis. Univariate analysis showed that the following factors were significantly related to postoperative survival: depth of invasion and lymph node metastasis, stage, lymphatic invasion, venous invasion and Osteopontin expression (*P*<0.05). Multivariate regression analysis indicated that depth of invasion, lymph node metastasis and venous invasion were independent prognostic factors, but that lymphatic invasion and Osteopontin expression were not independent prognostic factors.

## DISCUSSION

In this study, we immunohistochemically investigated the relationship between Osteopontin expression and clinicopathological factors, including prognosis, in ESCC. The expression of Osteopontin was observed in 48% of tumours. This was consistent with previous immunohistochemical studies on other carcinomas in which Osteopontin expression was detected in 33.3–70.0% (Ue *et al*, 1998; Zhang *et al*, 2001; Coppola *et al*, 2004).

In the present study, Osteopontin expression was significantly associated with lymph node metastasis and lymphatic invasion. In non-small cell lung cancer, the expression of Osteopontin in surgical specimens is significantly correlated with tumour size, lymph node metastasis and stage (Donati *et al*, 2005). In the ESCC, plasma Osteopontin was associated with lymph node metastasis (Shimada *et al*, 2005). In gastric cancer, the expression of Osteopontin in poorly differentiated tumours is also associated with lymphogenous metastasis (Ue *et al*, 1998). Taking these results together, it appears that the expression of Osteopontin in some human malignant tumours might be more associated with metastasis than with tumorigenesis.

The molecular mechanisms that define the role of Osteopontin in tumour metastasis have not been completely elucidated, although several mechanisms have been suggested. Previous studies showed that Osteopontin can support attachment for a variety of cell types and promote migration of tumour cells (Oates *et al*, 1997; Tuck *et al*, 2000). Osteopontin contains the cell attachment amino-acid sequence RGD (arginine–glycine–aspartic acid), which bind to the alpha vs beta 3 integrin heterodimer, play a role in cell adhesion (Xuan *et al*, 1995). In addition, Osteopontin bind several different cellular receptors, potentially allowing it to stimulate various signalling pathways and influence cellular events that may ultimately promote tumorgenesis, adhesion, migration and metastasis (Hu *et al*, 1995; Liaw *et al*, 1995; Weber *et al*, 1996; Al-Shami *et al*, 2005). The down-stream Osteopontin signals which interrupt the cell cycle, prevent apoptosis and promote cell survival are integral to tumour progression (Evan and Vousden, 2001). There may be especially signals that corresponded with lymph node metastasis in those signals that Osteopontin stimulate. In the present study, close relationship was found between Osteopontin expression and lymph node metastasis.

Concerning the survival analysis, sex, tumour depth, lymph node metastasis, stage, lymphatic invasion, venous invasion and Osteopontin expression were prognostic factors on univariate analysis. However, multivariate analysis revealed that only tumour depth, nodal metastasis and venous invasion were independent prognostic factors. It has previously been demonstrated that plasma Osteopontin in ESCC is associated with poor survival (Shimada *et al*, 2005). Expression of Osteopontin is also significantly associated with poor survival in stage I non-small cell lung cancer (Donati *et al*, 2005). These findings indicate that the Osteopontin overexpression in some tumours is correlated with poor prognosis, predominantly via lymph node metastasis. Although Osteopontin expression in ESCC was not found to be an independent prognostic factor in the present study, it might play an important role in promoting progression and migration to the lymphatic system. Thus, evaluation of Osteopontin expression using biopsy specimens before surgical therapy may be a new standard for appropriate lymph node dissection of ESCC.

In conclusion, we detected Osteopontin protein in ESCC and found this to be associated with lymph node metastasis, stage, lymphatic invasion, and prognosis. Osteopontin is therefore useful for predicting malignant properties. These findings suggested a possible role for Osteopontin expression level as a new diagnostic and prognostic biomarker for ESCC. Furthermore, understanding the biological function of Osteopontin expression in ESCC may help to determine its role in physiology of ESCC.

## Figures and Tables

**Table 1:** Relationship between Osteopontin expression and clinicopathological findings

		**Osteopontin**		
		**Positive**	**Negative**	
	**Total (*n*=175)**	***n*=84 (48.0%)**	***n*=91 (52.0%)**	***P*-value**
*Age*				
Mean		62.8	63.3	0.7041
SD^a^		8.0	9.8	
				
*Gender*				
Male	160	80 (87.9)	80 (95.2)	0.0775
Female	15	11 (12.1)	4 (4.8)	
				
*Tumour location*				
Upper	23	15 (16.5)	8 (9.5)	0.1611
Middle	92	50 (55.0)	42 (50.0)	
Lower	60	26 (28.5)	34 (40.5)	
				
*Histology*				
Well	62	29 (34.5)	33 (36.3)	0.5678
Moderate	85	39 (46.6)	46 (50.5)	
Poor	28	16 (19.1)	12 (13.8)	
				
*pT*				
pT1	58	24 (28.6)	34 (37.4)	0.4339
pT2	22	9 (10.7)	13 (14.3)	
pT3	66	35 (41.7)	31 (34.0)	
pT4	29	16 (19.0)	13 (14.3)	
				
*pN*				
pN0	78	27 (32.1)	51 (56.0)	0.0015
pN1	97	57 (77.9)	40 (44.0)	
				
*pM*				
pM0	131	57 (67.9)	74 (81.3)	0.1398
pM1	44	27 (32.1)	17 (18.7)	
				
*Stage*				
I	41	14 (16.7)	27 (29.7)	0.0333
IIA	31	12 (14.2)	19 (20.9)	
IIB	21	14 (16.7)	7 (7.7)	
III	38	17 (20.2)	21 (23.1)	
IV	44	27 (32.2)	17 (18.6)	
				
*Lymphatic invasion*				
Negative	66	25 (29.8)	41 (45.1)	0.0375
Positive	109	59 (70.2)	50 (54.9)	
				
*Venous invasion*				
Negative	125	52 (61.9)	66 (72.5)	0.1339
Positive	50	32 (38.1)	25 (27.5)	

a Standard deviation.

**Table 2:** Univariate analysis of prognostic factors in ESCC

**Variables**	***n***	**5-years survival rate (%)**	***P***
*Sex*			
Male	160	80.0	0.0139
Female	15	44.6	
			
*Tumour depth*			
pT1,2	80	74.8	<0.0001
pT3,4	95	24.3	
			
*Lymph node metastasis*			
Negative	78	75.4	<0.0001
Positive	97	15.8	
			
*Stage*			
I, II	93	75.9	<0.0001
III, IV	82	17.0	
			
*Lymphatic invasion*			
Negative	66	75.4	<0.0001
Positive	109	31.5	
			
*Venous invasion*			
Negative	125	59.8	<0.0001
Positive	50	24.7	
			
*Osteopontin*			
Positive	84	56.6	0.0354
Negative	91	39.2	

**Table 3:** Multivariate analyses of prognostic factors in ESCC

**Independent factors**	**Multivariate *P***	**Hazard ratio**	**95% Confidence interval**
*pT*			
1,2/3,4	<0.0001	4.024	2.428–6.671
			
*pN*			
Negative/positive	0.0210	2.192	1.125–4.270
			
*Lymphatic invasion*			
Negative/positive	0.0984	1.849	0.892–3.835
			
*Venous invasion*			
Negative/positive	0.3005	1.281	0.802–2.046
			
*Osteopontin expression*			
Negative/positive	0.2869	1.271	0.818–1.975
